# Draft Genome Sequence of Bacillus velezensis CE2, Which Genetically Encodes a Novel Multicomponent Lantibiotic

**DOI:** 10.1128/MRA.01420-18

**Published:** 2019-01-17

**Authors:** Emily Campbell, Michelle Gerst, B. Carol Huang, Nguyet Kong, Bart C. Weimer, Ahmed E. Yousef

**Affiliations:** a Department of Food Science and Technology, The Ohio State University, Columbus, Ohio, USA; b Department of Microbiology, The Ohio State University, Columbus, Ohio, USA; c School of Veterinary Medicine, 100K Pathogen Genome Project, University of California Davis, Davis, California, USA; Louisiana State University

## Abstract

Bacillus velezensis CE2 produces potent antimicrobial compound(s). The draft genome sequence of the strain reported here is 4.1 Mb with a G+C content of 46.1%. Whole-genome sequencing revealed that the strain genetically encodes a novel multicomponent lantibiotic, velezensicidin.

## Abstract (precis)

Bacillus velezensis CE2 produces potent antimicrobial compound(s). The draft genome sequence of the strain reported here is 4.1 Mb with a G+C content of 46.1%.

## ANNOUNCEMENT

A number of Bacillus velezensis strains were reported to produce antimicrobials (1 – 6). A high-throughput culture-based assay was used to screen soil microbiota for antimicrobial production (7). Using this method, B. velezensis CE2 was isolated and found to produce potent antimicrobial activity against Gram-positive bacteria.

The whole genome of B. velezensis CE2 was sequenced within the 100K Pathogen Genome Sequencing Project (8), as described previously (9). Briefly, genomic DNA (gDNA) was extracted and purified from B. velezensis CE2 culture, grown on MRS agar (Becton, Dickinson, Franklin Lakes, NJ) at 30°C for 24 h, using a Qiagen DNA genomic minikit (catalog number 51306). gDNA quality was determined, using *A*_260/280_ and *A*_260/230_ ratios, to be >1.5, and size characterization was done using an Agilent 2200 TapeStation system, as described previously (10). Libraries were constructed using a Kapa HyperPlus library preparation kit (catalog number KK8514) and indexed with Weimer 384 TS-LT DNA barcodes (Integrated DNA Technologies, Inc., Coralville, IA, USA). The resulting libraries were 350 to 450 bp. Library amplification was completed in eight cycles using the Kapa HiFi HotStart ReadyMix PCR kit, followed by cleanup with 1× solid-phase reversible immobilization (SPRI) magnetic beads (9). Final library quality control was done using an Agilent 2100 Bioanalyzer with a high-sensitivity DNA kit. Sequencing library concentration was determined by quantitative PCR (qPCR) using the Kapa library quantification kit with universal qPCR mix (catalog number KK4824) before indexing. Libraries were sequenced using an Illumina HiSeq X Ten instrument with a 150-bp paired end (PE150) read (Novagene, Sacramento, CA). For B. velezensis CE2, the total number of paired reads generated was 1,213,887. These were assembled using SPAdes version 3.11.1 (settings: k-mer sizes 21, 33, 55, and 77 with mismatch careful mode) (11), after PhiX and indexes were removed by NCBI’s contamination filter (VecScreen). After assembly, contigs were ordered using Bacillus velezensis FZB42 as a reference, with progressiveMAUVE (version 2.4.0) and applying the multiple genome alignment option (12). The genome contained 265 contigs, with a coverage of 44×. The maximum and minimum sizes of contigs were 558,902 bp and 225 bp, respectively, with an *N*_50_ value of 297,815. The genome was annotated by Rapid Annotations using Subsystems Technology (RAST) version 2.0 (default settings), and 4,438 coding regions and 88 rRNA were found (13).

BAGEL version 3 (default settings) was used to identify the lantibiotic gene cluster in the draft genome sequence (14). Two-component lantibiotic structural genes (*lanA*), two modification enzymes genes (*lanM*), and a transporter gene (*lanT*) were identified in the genome. To the best of our knowledge, the amino acid sequences encoded by the structural genes for the two-component lantibiotic were not reported elsewhere in published literature. The production of potent antimicrobial activity and presence of a novel lantibiotic structural gene indicate the potential usefulness of B. velezensis CE2 in food or agriculture.

### Data availability.

Assembled and raw reads can be found at the 100K Project BioProject (accession number PRJNA203445) in the Sequence Read Archive (https://www.ncbi.nlm.nih.gov/sra). The accession number for the DDBJ/ENA/GenBank assembly is RBZX00000000 and reads are in the SRA under the accession number SRR7965938. The sample information can be found under BioSample accession number SAMN10178429. The assembly version described here is version RBZX01000000.
